# Relationships of Dietary Histidine and Obesity in Northern Chinese Adults, an Internet-Based Cross-Sectional Study

**DOI:** 10.3390/nu8070420

**Published:** 2016-07-11

**Authors:** Yan-Chuan Li, Chun-Long Li, Jia-Yue Qi, Li-Na Huang, Dan Shi, Shan-Shan Du, Li-Yan Liu, Ren-Nan Feng, Chang-Hao Sun

**Affiliations:** 1 Department of Nutrition and Food Hygiene, School of Public Health, Harbin Medical University, No. 157 Baojian Road, Nangang District, Harbin 150086, China; liyanchuan2013@foxmail.com (Y.-C.L.); qijiayue678@163.com (J.-Y.Q.); shidanhmu@foxmail.com (D.S.); dushanshan1007@163.com (S.-S.D.); yanziliu2100@163.com (L.-Y.L.); 2 Department of General Surgery, The Second Affiliated Hospital of Harbin Medical University, Harbin 150086, China; chunlong81@163.com; 3 Liaoning Institute for Food Control, Shenyang 110015, China; lnspjcy@163.com

**Keywords:** dietary histidine, overweight/obesity, insulin resistance, inflammation, oxidative stress

## Abstract

Our previous studies have demonstrated that histidine supplementation significantly ameliorates inflammation and oxidative stress in obese women and high-fat diet-induced obese rats. However, the effects of dietary histidine on general population are not known. The objective of this Internet-based cross-sectional study was to evaluate the associations between dietary histidine and prevalence of overweight/obesity and abdominal obesity in northern Chinese population. A total of 2376 participants were randomly recruited and asked to finish our Internet-based dietary questionnaire for the Chinese (IDQC). Afterwards, 88 overweight/obese participants were randomly selected to explore the possible mechanism. Compared with healthy controls, dietary histidine was significantly lower in overweight (*p* < 0.05) and obese (*p* < 0.01) participants of both sexes. Dietary histidine was inversely associated with body mass index (BMI), waist circumference (WC) and blood pressure in overall population and stronger associations were observed in women and overweight/obese participants. Higher dietary histidine was associated with lower prevalence of overweight/obesity and abdominal obesity, especially in women. Further studies indicated that higher dietary histidine was associated with lower fasting blood glucose (FBG), homeostasis model assessment of insulin resistance (HOMA-IR), 2-h postprandial glucose (2 h-PG), tumor necrosis factor-α (TNF-α), interleukin-1β (IL-1β), interleukin-6 (IL-6), C-reactive protein (CRP), malonaldehyde (MDA) and vaspin and higher glutathione peroxidase (GSH-Px), superoxide dismutase (SOD) and adiponectin of overweight/obese individuals of both sexes. In conclusion, higher dietary histidine is inversely associated with energy intake, status of insulin resistance, inflammation and oxidative stress in overweight/obese participants and lower prevalence of overweight/obesity in northern Chinese adults.

## 1. Introduction

The rising prevalence of overweight and obesity has become a major global health challenge [1]. Obesity is a major risk factor for a series of metabolic disorders and chronic diseases, including insulin resistance, metabolic syndrome, type 2 diabetes, hypertension, cardiovascular diseases and certain cancer [2,3,4,5]. Accumulating evidence has proved that nutritional factors are strongly associated with the development, treatment and prevention of chronic diseases, such as obesity, hyperlipidemia, diabetes mellitus and cardiovascular diseases [6,7,8,9]. Therefore, it is urgent to improve etiologic research on diet by investigating nutrient and food intakes and reasonable dietary guidelines should be proposed by the government.

Histidine, a precursor for the synthesis of histamine, is abundant in red meat and fish and is an important amino acid for humans. It has been reported that lower plasma concentration of histidine is associated with protein-energy wasting, inflammation and oxidative stress in chronic kidney disease patients [10]. Previous animal studies confirmed that histidine supplementation could reduce body weight and ameliorates inflammation and oxidative stress of adipose tissue in a high-fat diet induced female obese rat model [11]. Moreover, dietary histidine also suppresses food intake and fat accumulation in rats [12]. In our previous study, we had found that serum histidine concentrations in obese women were significantly lower than those in non-obese women and had negative relationships with inflammation and oxidative stress [13]. The recovery of serum histidine concentrations through histidine supplementation could improve insulin resistance, reduce body mass index (BMI) and fat mass, and suppress inflammation and oxidative stress in obese women [14]. Only one cross-sectional study in Japanese adolescents showed a significantly negative correlation between energy intake and ratio of histidine to protein intake [15].

Although the central roles of dietary histidine for regulation of energy intake have already been established in animal and in vitro studies, to our knowledge, no study has analyzed the associations between dietary histidine and prevalence of obesity in general population. Moreover, whether chronic intake of dietary histidine is associated with insulin resistance, inflammation and oxidative stress in overweight/obese individuals is still unclear. The contribution of dietary histidine is not yet fully confirmed. Therefore, we assessed dietary histidine intakes of 2376 participants and examined relationships between dietary histidine and obesity in this population in this study. Furthermore, a subgroup study of 88 overweight/obese subjects (44 men and 44 women) was performed to explore the possible mechanism of the anti-obesity effect of histidine.

## 2. Materials and Methods

### 2.1. Development and Validation of the IDQC

To investigate dietary habits accurately, many methods for dietary assessment have been used by researchers. The food frequency questionnaire (FFQ) provides us a convenient and economic method to assess the general dietary intakes in a large population [16]. However, traditional face-to-face FFQs are impossible to conduct in a large population during a short period as the face-to-face FFQs always consume much time [17]. Previously, a convenient tool named Internet-based dietary questionnaire for the Chinese (IDQC) has been designed and validated at Harbin Medical University previously by experts of nutrition, epidemiology and bio-statistics [18].

Commonly eaten foods were divided into 16 categories (i.e., grains, potatoes, legumes, vegetables, fungus, fruits, seeds and nuts, livestock, poultry, dairy, eggs, fish, snacks, sugar, condiments, and beverages). Reference images of each food item’s weight/volume were created as references to assist the participants in making accurate estimation of food portions. Each participant had to choose the frequency and amount of each subtype of food groups. Finally, the questionnaire was uploaded to a secure website, which is free of access [19].

The IDQC has been validated as a convenient dietary assessment tool in our previous study [18]. Briefly, 644 recruited participants had completed the IDQC, the intakes of the food groups and nutrients in the IDQC were validated against those in the 3-day dietary records. The IDQC has been confirmed as an accurate tool for dietary assessment, because it has good consistency to results of 3-day dietary records. Therefore, the IDQC can be used for dietary assessment in large population.

### 2.2. Participants, Exclusive Criteria, Power Calculation and Study Design

**Figure 1:**
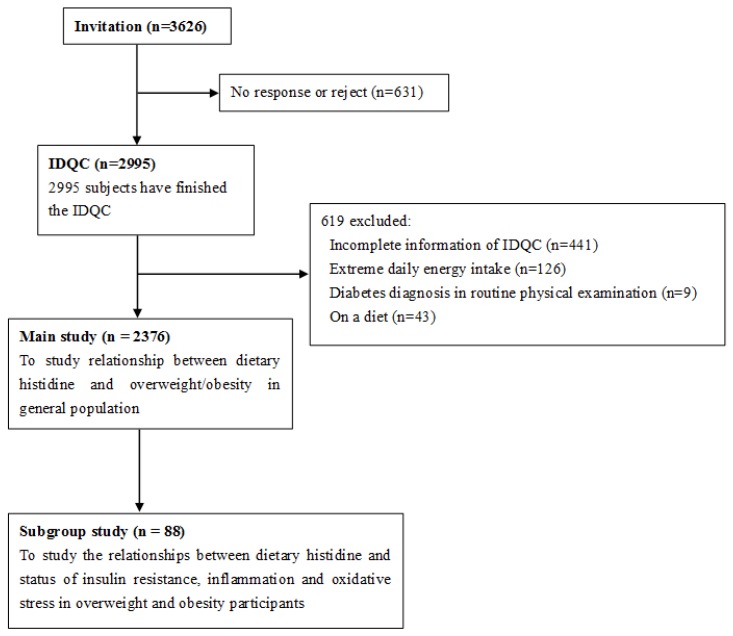
Flow of the study population.

We randomly invited 3626 participants from the Health Examination Center of the Second Affiliated Hospital of Harbin Medical University. Of the 3626 invited participants, 2995 agreed to participate in our study, register an account and finish the IDQC. Firstly, online demographic questionnaires were completed by all participants; the details of which were provided in our previous published manuscript [20]. The exclusive criteria were as follows: (1) incomplete information on the IDQC; (2) extreme daily energy intake (<800 kcal (3349 kJ) or >5000 kcal (20,934 kJ) for males; <600 kcal (2512 kJ) or >4000 kcal (16,747 kJ) for females); (3) low BMI (BMI < 18.5); and (4) diabetes diagnosis or being on a diet in the past 6 months. In total, 619 were excluded for the above reasons (Figure 1).

For power calculation, considering the 9.9 cm standard deviation of waist circumference (WC) in the overall population, a sample size of 594 in each quartile will be sufficient to detect a difference of 2.4 cm between quartiles. Moreover, this sample size is also sufficient to detect a difference of 1.0 kg/m^2^ in BMI, at 99% power and 5% level of significance.

To explore possible mechanisms of the anti-obesity effect of histidine, 88 overweight/obese subjects were randomly selected by stratified sampling for subgroup study. Except for dietary histidine (% protein), body weight, BMI, WC, and drinking and smoking habits, there is no difference between included participants and excluded participants. Characteristics are presented in Table S1. An oral glucose tolerance test (OGTT) was performed and metabolic profile, status of inflammation and oxidative stress were also examined. A flow chart of the study population is shown in Figure 1. This study was conducted according to the guidelines of the Declaration of Helsinki and all procedures involving humans were approved by the Human Research Ethics Committee of the Harbin Medical University (approval code [2015]006). Online informed consent was obtained from all participants.

### 2.3. Estimation of Dietary Nutrients Intake

After the online demographic questionnaire, all participants were asked to complete the IDQC for the past 4 months, and details of the IDQC are provided in our previous published manuscript [20]. The intake detail (frequency and amount) of each kind of food was obtained and average daily intakes of all nutrients were then calculated according to the China Food Composition Tables [21]. China Food Composition Tables is an important reference book for researchers of nutrition and public health of China. In this book, commonly eaten Chinese foods are evaluated and average nutrient contents (including energy, macro nutrients, trace elements, amino acids, and fatty acids) of these foods are measured by researchers and provided in the book. In our study, the histidine amount is total dietary source histidine (including protein, peptide and free form of histidine).

### 2.4. Anthropometric Measurements

All participants were asked to stand on an anthropometer without heavy clothing and shoes. Body weight and height were measured to the nearest 0.1 kg and 0.1 cm. For WC measurement, the same standard was applied for each individual. WC was measured using a flexible anthropometric tape on the horizontal plane between the lowest rib and the iliac crest, to the nearest 0.1 cm. Blood pressure was measured using a standard mercury sphygmomanometer. Each participant was seated comfortably for 10 min in a quiet room. Korotkoff sounds I and V were criteria for systolic blood pressure (SBP) and diastolic blood pressure (DBP), respectively. SBP and DBP were measured twice and the mean values were calculated. All anthropometric measurements were performed by standardized and trained personnel and have good reproducibility.

### 2.5. Definition of Overweight/Obesity and Abdominal Obesity

BMI was defined as weight in kilograms divided by the square of height in meters (kg/m^2^). As this study was performed in Chinese population, the BMI cut-off points of Chinese subjects (overweight: 24.0–27.9; obesity ≥ 28.0) were used [22]. Abdominal obesity was defined as WC ≥ 85 cm in men and WC ≥ 80 cm in women, according to the 2006 Guidelines on Preservation and Control Overweight and Obesity in Chinese Adults classification [23].

### 2.6. Collection and Laboratory Analysis of Serum

Eighty-eight participants were instructed to fast overnight (more than 12 h) and fasting blood samples were collected in the morning. After fasting blood samples were obtained, each participant was asked to take 75 g glucose (dissolved in 200 mL of warm water), and after 2 h, postprandial blood samples were obtained. Blood samples were centrifuged at 3000 rpm for 15 min to obtain serum and serum were stored at −80 °C immediately. Fasting and 2 h-postprandial blood glucose levels were measured with a hand-held glucose monitoring system (One-Touch Ultra 2; Life Scan, Milpitas, CA, USA). Serum insulin concentration was measured by ROCHE Elecsys 2010 Chemiluminescence Immune Analyzer (Roche Diagnostics, Mannheim, Germany). Fasting and 2 h-postprandial serum triglyceride (TG,) total cholesterol (TC), high destiny lipoprotein (HDL) and low destiny protein (LDL) were determined using a ROCHE Modular P800 Automatic Biochemical Analyzer (Roche Diagnostics, Mannheim, Germany). Homeostasis model assessment of insulin resistance (HOMA-IR) index was calculated as previously described [24]. Serum tumor necrosis factor-α (TNF-α), interleukin-1β (IL-1β), interleukin-6 (IL-6), C-reactive protein (CRP), adiponectin and vaspin concentrations were assayed using ELISA with commercial kits (TNF-α, IL-1β, IL-6, and vaspin, catalog number CSB-E04740h, CSB-E08053h, CSB-E04638h, CSB-E09771h, Cusabio, China; CRP, catalog number BC-1119, Biocheck, USA; adiponectin, catalog number ab99968, Abcam, UK). Serum superoxide dismutase (SOD), glutathione peroxidase (GSH-Px), malonaldehyde (MDA) were measured with enzymatic methods using commercial kits (Jiancheng Technology, Nanjing, China). Coefficients of variations (CVs) of lab measurements are presented in Supplementary Materials.

### 2.7. Statistical Analysis

For descriptive statistics, means and SDs (or frequencies and percentages) were calculated across BMI categories (normal weight: BMI < 24.0; overweight: 24.0–27.9; obese: ≥28.0) and compared using one-way analysis of variance (one-way ANOVA) or chi-square test, as appropriate. Dietary histidine of different BMI categories was further analyzed by means of analysis of covariance (ANCOVA).

Considering the high correlation between histidine and total protein intake (*r* = 0.925), and that nutrient densities can be used to reduce the likelihood of multicollinearity [25], we used histidine (percent total protein intake) in our study. Bivariate correlation analysis (without variants adjusted) and partial correlation analysis was performed to assess the association of branched-chain amino acid (BCAA) and BMI, WC, SBP and DBP (adjusting for age, dietary carbohydrate, fat, protein, cholesterol and fiber intake), fasting blood glucose (FBG), insulin, HOMA-IR, TC, TG, HDL, LDL, 2-h postprandial glucose (2 h-PG), 2 h-insulin, 2 h-TC, 2 h-TG, 2 h-HDL, 2 h-LDL, GSH-Px, SOD, MDA, TNF-α, IL-1β, IL-6, CRP, adiponectin and vaspin (adjusting for age, BMI, dietary carbohydrate, fat, protein, cholesterol and fiber intake).

To estimate the Odds ratio (OR) and 95% confidence interval (CI) of overweight/obesity and abdominal obesity between quartiles of dietary histidine, logistic regression model was used. Age, education, income, labor, exercise status, dietary carbohydrate, fat, protein, cholesterol, fiber intake and smoking habits in three models. The statistical analyses were carried out using SAS software (version 9.1; SAS Institute, Cary, NC, USA). By statistician and *p* < 0.05 was considered statistically significant.

## 3. Results

### 3.1. Descriptive Statistics

The characteristics of participants by dietary histidine quartiles are summarized in Table 1. Compared with those in the first quartile of dietary histidine, participants of the 4th quartile were more likely to be youthful and had lower body weight, BMI, WC, and higher percentage of women. The highest prevalence of current smoking was observed in the 2nd quartile of dietary histidine. Income, education, labor and physical exercise status were also different between histidine quartiles (all *p* < 0.05). SBP and DBP of the 4th quartile were also significantly lower than those in the 1st quartile of dietary histidine. The prevalence of overweight/obesity was 37.4% and the prevalence of abdominal obesity was 28.5% among our participants.

For dietary intake, total protein, total amino acids, total fat, cholesterol and fiber of the 3rd and 4th quartiles were significantly higher than the 1st quartile. Dietary carbohydrates of the 1st quartile was significantly higher than the 3rd and 4th quartiles. Total energy intake was also different between histidine quartiles (*p* = 0.048).

### 3.2. Dietary Histidine of Overweight and Obesity Participants Was Lower than Healthy Controls

**Figure 2:**
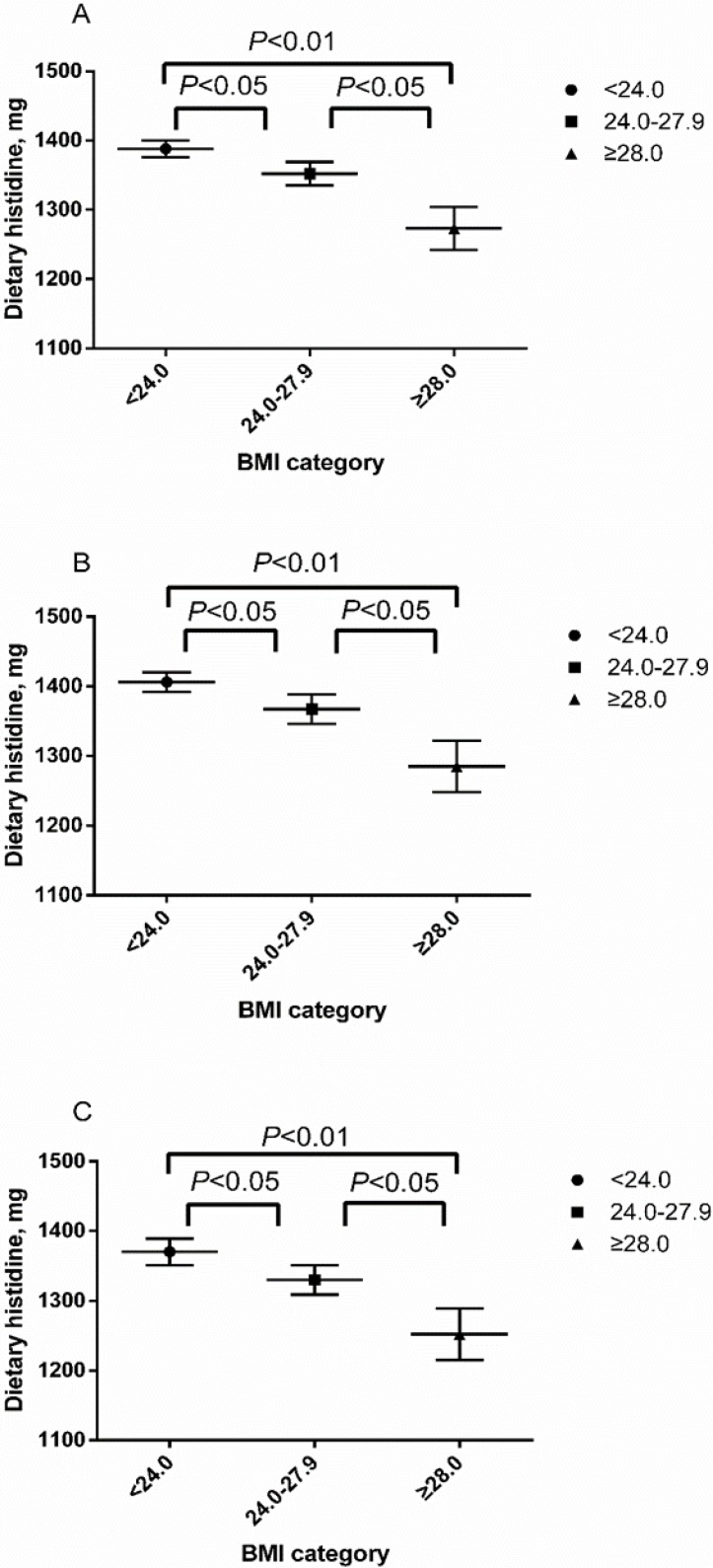
Dietary histidine of different body mass index (BMI) categories: (**A**) the overall population; (**B**) male participants; and (**C**) female participants. Confounding factors such as age, labor, exercise status, dietary carbohydrate, fat, protein, fiber and cholesterol intake and smoking habits were controlled.

We firstly performed this comparison to find the differences in dietary histidine among three BMI categories (normal weight < 24.0, overweight 24.0–28.0, and obesity ≥ 28.0). In the overall male and female populations, dietary histidine of overweight and obese groups were all significantly lower than normal group (*p* < 0.05 between normal and overweight group, *p* < 0.01 between normal and obese group). Moreover, dietary histidine of obese group was significantly lower than overweight group (*p* < 0.05). Confounding factors such as age, education, income, labor, exercise status, dietary carbohydrate, fat, protein, fiber and cholesterol intake and smoking habits were controlled in this study (Figure 2).

### 3.3. Correlations between Dietary Histidine and Body Weight, BMI, WC, SBP and DBP

Bivariate correlation analysis and partial correlation analysis was used to examine these associations of dietary histidine with body weight, BMI, WC, SBP and DBP. Potential covariates (age, dietary carbohydrate, fat, protein, fiber and cholesterol intake) were adjusted in the partial correlation analysis. As shown in Table 2, we found negative correlations between dietary histidine and body weight, BMI, WC, SBP and DBP (*r* = −0.076, −0.129, −0.132, −0.106 and −0.092, respectively, all *p* < 0.01). Because of higher histidine intake in males (male: 1.6 ± 0.8 g vs. female: 1.3 ± 0.6 g, *p* < 0.01), we examined these associations in both gender, the significance was stronger in women. Furthermore, considering the higher histidine intake in normal BMI participants (Figure 2), we examined these associations in different BMI categories. We found negative correlations between dietary histidine and BMI, WC, SBP and DBP in overweight and obese participants, but no significant correlation of dietary histidine and BMI, WC, and DBP were observed in normal BMI participants.

### 3.4. Higher Dietary Histidine Intake Is Associated with Lower Prevalence of Overweight/Obesity and Abdominal Obesity in Northern Chinese

With adjustment for potential dietary and non-dietary cofounders (age, gender; income; education; labor; physical exercise; dietary carbohydrate, fat, protein, fiber and cholesterol intake; and smoking status), higher dietary histidine was inversely associated with prevalence of overweight/obesity and Abdominal obesity. As shown in Table 3 and Table 4, compared with the 1st quartile, the multivariable-adjusted ORs of overweight/obesity for the 3rd and 4th quartile were 0.745 (0.572, 0.969) and 0.650 (0.482, 0.876), respectively (all *p* < 0.05). After being stratified by gender, the significance still exists in the 4th quartile of men and the 2nd, 3rd and 4th quartile of women (all *p* < 0.05). For abdominal obesity, the multivariable-adjusted ORs for the 2nd, 3rd and 4th quartile were 0.716 (0.539, 0.952), 0.809 (0.597, 0.995) and 0.754 (0.545, 0.943), respectively (all *p* < 0.05). After being stratified by gender, the significance is stronger in women (all *p* < 0.01) but no significant correlation was observed in men.

### 3.5. Correlations between Dietary Histidine and Status of Insulin Resistance, Inflammation and Oxidative Stress in Overweight/Obese Participants

Considering the stronger correlations between dietary histidine and BMI and WC in overweight/obese participants, 88 overweight/obese individuals were selected for subgroup study to explore the possible mechanism. Characteristics of subgroup study population are provided in Table S1. Overweight and obesity are always accompanied by abnormal postprandial metabolism, chronic low-grade inflammation and oxidative stress [20]. Therefore, serum biochemistry indexes and biomarkers of inflammation and oxidative stress were measured. Partial correlation analysis was used to examine these associations of dietary histidine with status of insulin resistance, inflammation and oxidative stress, independent of total energy intake. Potential covariates (age, BMI, dietary carbohydrate, fat, protein, fiber and cholesterol intake) were adjusted in the model. As shown in Table 5, higher dietary histidine is associated with lower FBG, HOMA-IR, 2 h-PG, MDA, TNF-α, IL-1β, IL-6, CRP, MDA and vaspin and higher GSH-Px, SOD and adiponectin of overweight/obese individuals of both sexes (all *p* < 0.05).

## 4. Discussion

This is the first Internet-based cross-sectional study to investigate associations between dietary histidine and prevalence of overweight/obesity and abdominal obesity in northern Chinese adults. We firstly reported that higher dietary histidine intake was associated with lower prevalence of overweight/obesity and abdominal obesity and lower BMI, waist circumference and blood pressure in northern Chinese population, which was firstly found using an Internet-based FFQ study. Moreover, we firstly reported that in overweight/obese individuals, higher dietary histidine is associated with lower FBG, HOMA-IR, 2 h-PG, MDA, TNF-α, IL-1β, IL-6, CRP, MDA and vaspin and higher GSH-Px, SOD and adiponectin.

In this study, we firstly found that dietary histidine is inversely associated with SBP and DBP in northern Chinese population, but no direct evidence was found to support our finding. Only one cross-sectional study reported a positive association between histidine and blood pressure in adolescents [25]. However, the relationship between obesity and hypertension is well established and the mechanisms through which obesity directly causes hypertension are still an area that requires more research [26]. The negative association between dietary histidine and blood pressure can be explained by anti-obesity effect of histidine.

Insulin resistance is the central feature of the metabolic syndrome and is considered as a critical link between obesity and many chronic diseases [27]. To effectively control these chronic diseases, the positive prevention of insulin resistance in obese individuals is extremely important. It has been reported that histidine supplementation could improve insulin resistance in obese women [14]. In the present study, we firstly found that dietary histidine is inversely associated with FBG, HOMA-IR and 2 h-PG in obese individuals, but no significant correlation between dietary histidine and fasting/postprandial insulin was found. These results indicated that dietary histidine also might improve insulin resistance in overweight and obese individuals. However, limited sample size may influence this effect and further studies are needed to confirm this association in normal weight individuals.

It has been confirmed that concentrations of pro-inflammatory cytokines such as TNF-α, IL-6 and CRP are elevated in obese subjects [28] and elevated TNF-α, IL-6 are associated with obesity related insulin resistance [29]. CRP is the most important inflammation biomarker in humans; it is elevated in status of systemic inflammation and is also related to insulin resistance and metabolic syndrome [30]. Therefore, the suppression of pro-inflammation cytokines is considered as an effective strategy for reducing the risk of obesity-related diseases. In animal models of diabetes, histidine supplementation could reduce the levels of IL-6, TNF-α and CRP [31]. Our previous studies in a high-fat diet induced female obese rat model also confirmed that histidine supplementation could ameliorate inflammation and oxidative stress of adipose tissue [11]. In an in vitro study, Son et al. [32] reported that histidine inhibited the hydrogen peroxide- (H_2_O_2_-) and TNF-α-induced IL-8 secretion at the transcriptional level in intestinal epithelial cells. In a previous study, we had found lower serum histidine concentrations in obese women than non-obese women. In obese women, serum histidine was negatively associated with inflammation and oxidative stress [13]. Histidine supplementation could improve IR, reduce BMI, fat mass and suppress inflammation and oxidative stress in obese women [14]. In the present study, we firstly found that dietary histidine was inversely associated with TNF-α, IL-1β, IL-6 and CRP, which indicated that dietary histidine also might improve inflammation in overweight and obese individuals. However, this correlation in normal weight individuals is unclear.

Adiponectin is a novel adipocytokine that has been suggested to play a role in the development of insulin resistance and atherosclerosis [33]. Circulating adiponectin levels were decreased in parallel with the development of insulin resistance in rhesus monkeys [34], which indicates that reduced plasma adiponectin levels might play a role in the progress of insulin resistance. In the present study, we also found positive correlation between dietary histidine and serum adiponectin in overweight/obese induviduals. Dietary histidine may increase secretion of adiponectin by suppressing inflammation and oxidative stress in white adipose tissue. Further studies are needed to verify this correlation in normal weight individuals.

Vaspin (visceral adipose tissue-derived serpin) was firstly found in an abdominal obesity with T2DM animal model and was shown as a new adipocytokine to influence insulin sensitivity of white adipose tissues in obese rats. Interestingly, the serum level of vaspin shows increasing trend in prediabetic stage, but decreased with the development of diabetes along with a sharp body weight loss [35]. Circulating vaspin is strongly associated with BMI and insulin sensitivity [36]. A meta-analysis also reporting vaspin levels in subjects with obesity and type 2 diabetes mellitus was higher than healthy controls [37]. In the present study, we firstly found that dietary histidine was inversely associated with serum vaspin in overweight/obese participants. However, the mechanism is unknown.

Oxidative stress is considered as one main cause of insulin resistance and many chronic diseases are characterized by excessive oxidative stress [38]. Therefore, the suppression of oxidative stress is an effective strategy for reducing the risk of obesity-related diseases. Previous study found that the concentrations of SOD in the serum and the mRNA expression of copper zinc superoxide dismutase (CuZnSOD) in the white adipose tissue were increased and the concentrations of MDA in the serum were decreased by histidine supplementation in high-fat diet-induced female obese rat model [11]. In the present study, we quantified three oxidative biomarkers, GSH-Px, SOD and MDA, and the results revealed that the concentrations of GSH-Px and SOD in the serum was positively associated with dietary histidine and the concentrations MDA in the serum was inversely associated with dietary histidine, which provides more evidence that histidine is a potential antioxidant in obese individuals.

For the gender and BMI difference of histidine in our study, we found that negative correlations between dietary histidine and BMI and WC were stronger in overweight and obese participants. Moreover, the association between dietary histidine and overweight/obesity and abdominal obesity is much stronger in women. Several potential mechanisms might account for this finding. In this study, lower histidine intake was observed in female participants and overweight/obese individuals, which may lead to lower serum histidine and stronger response to dietary histidine. Moreover, previous studies found that women were more sensitive to dietary histidine and energy intake than men [15,39], but the mechanisms are still unclear and further studies are needed.

The primary limitation of this study is the cross-sectional and retrospective design. We must acknowledge that reverse causality may be exists as cross-sectional studies were insufficient to confirm the causal relationship between dietary factors and occurrence of disease [17]. Long term cohort studies for this issue are needed to verify the casual relationship; Secondly, current study was only performed in a northern Chinese population. Consider of the large population and vast territory of China, we must be rigorous when extrapolating this result to the general population. Moreover, beneficial effects of dietary histidine such as lower fasting blood glucose, insulin resistance, inflammation and oxidative stress may be associated with histidine peptides (carnosine, anserine and balenine), which has been shown in recent studies of carnosine supplementation in animals [40] and more recently in humans [41]. However, in our study, data of dietary histidine peptides were lacking. In spite of these limitations, this study provides a lot of practical significance, especially in nutritional counseling. As nutrition is important for prevention of obesity and reduction of excess body weight, reasonable dietary intervention should be useful for the prevention of overweight/obesity. For overweight/obese clients, advice of consuming more histidine-rich foods may be useful for weight control.

## 5. Conclusions

In summary, our study adds evidence supporting the inverse associations between dietary histidine and obesity, possibly through reduction of energy intake and improvements of insulin resistance, inflammation and oxidative stress. To confirm the casual associations, long-term cohort studies of this issue are needed.

## Figures and Tables

**Table 1:** Characteristics and dietary intake of participants by quartile of histidine intake.

	Quartiles of Histidine (% Total Protein Intake)				*p*
	1	2	3	4	
Histidine, % total protein intake	<1.38	1.38–1.59	1.59–1.77	>1.77	
Participants, *n*	594	594	594	594	
BMI categories					<0.001
<24.0, *n* (%)	327 (55.1)	352 (59.3)	384 (64.6)	424 (71.4)	
24.0–28.0, *n* (%)	191 (32.2)	182 (30.6)	165 (27.8)	141 (23.7)	
≥28.0, *n* (%)	76 (12.8)	60 (10.1)	45 (7.6)	29 (4.9)	
Abdominal obesity					0.020
Yes, *n* (%)	191 (32.1)	174 (29.3)	168 (28.3)	143 (24.1)	
No, *n* (%)	403 (67.9)	420 (70.7)	426 (71.7)	451 (75.9)	
Age, year	34.7 ± 14.9	34.6 ± 16.3	33.3 ± 15.5	29.9 ± 14.5	<0.001
Gender					0.018
Men, *n* (%)	319 (53.7)	296 (49.8)	266 (44.8)	283 (47.6)	
Women, *n* (%)	275 (46.3)	298 (50.2)	328 (55.2)	311 (52.4)	
Body weight, kg	67.6 ± 12.5	66.1 ± 11.6	65.4 ± 11.3	64.2 ± 11.4	<0.001
BMI, kg/m^2^	23.8 ± 3.7	23.3 ± 3.3	23.2 ± 3.3	22.8 ± 3.1	<0.001
WC, cm	80.6 ± 10.3	79.2 ± 9.7	79.0 ± 9.5	78.2 ± 9.8	<0.001
Income per month					<0.001
<2000 yuan, *n* (%)	355 (59.8)	366 (61.6)	374 (63.0)	442 (74.4)	
2000–5000 yuan, *n* (%)	223 (37.5)	207 (34.8)	203 (34.2)	123 (20.7)	
≥5000 yuan, *n* (%)	16 (2.7)	21 (3.5)	17 (2.9)	29 (4.9)	
Education					0.021
Under college, *n* (%)	175 (29.5)	145 (24.4)	132 (22.2)	122 (20.5)	
Bachelor, *n* (%)	398 (67.0)	424 (71.4)	437 (73.6)	449 (75.6)	
Master or doctor, *n* (%)	21 (3.5)	25 (4.2)	25 (4.2)	23 (3.9)	
Labor					<0.001
Light, *n* (%)	164 (27.6)	179 (30.1)	149 (25.1)	135 (22.7)	
Medium, *n* (%)	391 (65.8)	397 (66.8)	430 (72.4)	453 (76.3)	
Heavy, *n* (%)	39 (6.6)	18 (3.0)	15 (2.5)	6 (1.0)	
Exercise					<0.001
<10 h/week, *n* (%)	281 (47.3)	223 (3.8)	182 (30.6)	137 (23.1)	
10–20 h/week, *n* (%)	300 (50.5)	315 (53.0)	317 (53.4)	334 (56.2)	
≥20 h/week, *n* (%)	13 (2.2)	56 (9.4)	95 (16.0)	123 (20.7)	
Smoking					0.046
Non-smoker, *n* (%)	493 (83.0)	494 (83.2)	530 (89.2)	507 (85.4)	
Current smoker, *n* (%)	73 (12.3)	75 (12.6)	49 (8.2)	61 (10.3)	
Quit smoking, *n* (%)	28 (4.7)	25 (4.2)	15 (2.5)	26 (4.4)	
Drinking					0.44
Non-drinker, *n* (%)	475 (80.0)	489 (82.3)	495 (83.7)	480 (80.8)	
Current drinker, *n* (%)	119 (20.0)	105 (17.7)	99 (16.7)	114 (19.2)	
SBP (mmHg)	119.1 ± 12.9	119.0 ± 14.1	117.7 ± 12.4	115.1 ± 11.4	<0.001
DBP (mmHg)	78.9 ± 8.7	78.7 ± 9.1	77.9 ± 8.9	76.2 ± 8.4	<0.001
Dietary intakes					
Energy, kcal/day	2448.4 ± 824.5	2534.2 ± 867.6	2430.5 ± 865.5	2399.9 ± 896.0	0.048
Total protein, g/day	79.7 ± 30.5	92.7 ± 35.2	93.8 ± 37.9	95.0 ± 41.7	<0.001
Total amino acids, g/day	36.9 ± 16.8	54.2 ± 20.7	61.3 ± 24.7	69.8 ± 30.5	<0.001
Histidine, g/day	0.9 ± 0.4	1.4 ± 0.5	1.6 ± 0.6	1.8 ± 0.8	<0.001
Total fat, g/day	60.8 ± 28.2	78.5 ± 35.3	83.4 ± 37.6	81.2 ± 43.0	<0.001
Total carbohydrate, g/day	406.9 ± 146.3	382.5 ± 137.9	345.2 ± 133.4	341.9 ± 141.9	<0.001
Cholesterol, mg/day	405.7 ± 309.9	495.8 ± 338.9	489.6 ± 309.6	435.8 ± 349.0	<0.001
Fiber, g/day	15.7 ± 7.6	20.9 ± 10.6	21.7 ± 11.1	22.6 ± 13.0	<0.001

Abbreviations: BMI, body mass index; WC, waist circumference; SBP, systolic blood pressure; DBP, diastolic blood pressure; *p* are for differences across quartiles of histidine intake. Data are expressed as mean ± SD or frequencies and percentages, as appropriate.

**Table 2:** Correlations between dietary histidine and energy intake, body weight, BMI, WC and blood pressure.

	Unadjusted		Adjusted *			Unadjusted		Adjusted *	
	*r*	*p*	*r*	*p*		*r*	*p*	*r*	*p*
Overall					Normal BMI				
Energy intake	−0.031	NS	−0.053	<0.05	Energy intake	−0.032	NS	−0.027	NS
Body weight	−0.093	<0.001	−0.076	<0.01	Body weight	−0.042	NS	−0.029	NS
BMI	−0.126	<0.001	−0.129	<0.001	BMI	−0.046	NS	−0.037	NS
WC	−0.127	<0.001	−0.132	<0.001	WC	−0.061	NS	−0.071	NS
SBP	−0.119	<0.001	−0.106	<0.001	SBP	−0.109	<0.001	−0.056	<0.05
DBP	−0.104	<0.001	−0.092	<0.001	DBP	−0.079	<0.01	−0.052	NS
Men					Overweight				
Energy intake	−0.022	NS	−0.034	NS	Energy intake	−0.055	NS	−0.058	NS
Body weight	−0.061	<0.05	−0.064	<0.05	Body weight	−0.094	NS	−0.042	NS
BMI	−0.102	<0.01	−0.095	<0.05	BMI	−0.137	<0.001	−0.133	<0.001
WC	−0.096	<0.05	−0.093	<0.05	WC	−0.114	<0.05	−0.125	<0.05
SBP	−0.143	<0.001	−0.076	<0.05	SBP	−0.104	<0.01	−0.097	<0.05
DBP	−0.112	<0.001	−0.086	<0.01	DBP	−0.112	<0.01	−0.092	<0.05
Women					Obesity				
Energy intake	−0.065	<0.05	−0.056	<0.05	Energy intake	−0.068	<0.05	−0.073	<0.05
Body weight	−0.096	<0.05	−0.088	<0.01	Body weight	−0.119	<0.05	−0.104	<0.05
BMI	−0.136	<0.001	−0.147	<0.001	BMI	−0.131	<0.01	−0.147	<0.001
WC	−0.138	<0.01	−0.153	<0.001	WC	−0.143	<0.01	−0.142	<0.01
SBP	−0.066	<0.05	0.113	<0.01	SBP	−0.117	<0.01	−0.113	<0.05
DBP	−0.065	<0.05	−0.109	<0.01	DBP	−0.143	<0.05	−0.131	<0.05

Abbreviations: BMI, body mass index; WC, waist circumference; SBP, systolic blood pressure; DBP, diastolic blood pressure; NS, no significance. * Adjusting for dietary carbohydrate, fat, protein, fiber and cholesterol intake.

**Table 3:** Multivariable-adjusted Odds ratio (OR) and 95% confidence interval (CI) of overweight/obesity by quartile of dietary histidine.

All				
Histidine Quartiles	Quartile 1	Quartile 2	Quartile 3	Quartile 4
Dietary histidine (% protein)	<1.38	1.38–1.59	1.59–1.77	>1.77
Participants, *n*	594	594	594	594
Overweight/obesity, *n* (%)	267 (44.9)	242 (40.7)	210 (35.4)	170 (28.6)
Crude	1	0.768 (0.610, 0.968) *	0.736 (0.584, 0.928) *	0.537 (0.423, 0.681) **
Model 1	1	0.772 (0.602, 0.990) *	0.752 (0.586, 0.966) *	0.646 (0.500, 0.834) **
Model 2	1	0.777 (0.587, 1.029)	0.744 (0.572, 0.968) *	0.649 (0.481, 0.875) **
Model 3	1	0.772 (0.583, 1.023)	0.745 (0.572, 0.969) *	0.650 (0.482, 0.876) **
**Men**				
**Histidine Quartiles**	**Quartile 1**	**Quartile 2**	**Quartile 3**	**Quartile 4**
Dietary histidine (% protein)	<1.35	1.35–1.57	1.57–1.76	>1.76
Participants, *n*	291	291	291	291
Overweight/obesity, *n* (%)	132 (45.4)	117 (40.2)	98 (33.7)	85 (29.2)
Crude	1	0.926 (0.671, 1.278)	0.826 (0.592, 1.154)	0.608 (0.434, 0.853) **
Model 1	1	0.919 (0.655, 1.289)	0.843 (0.594, 1.197)	0.599 (0.490, 0.998) *
Model 2	1	0.973 (0.676, 1.388)	0.828 (0.561, 1.288)	0.578 (0.468, 0.991) *
Model 3	1	0.969 (0.672, 1.396)	0.838 (0.577, 1.302)	0.575 (0.456, 0.988) *
**Women**				
**Histidine Quartiles**	**Quartile 1**	**Quartile 2**	**Quartile 3**	**Quartile 4**
Dietary histidine (% protein)	<1.40	1.40–1.61	1.61–1.77	>1.77
Participants, *n*	303	303	303	303
Overweight/obesity, *n* (%)	135 (44.6)	125 (41.3)	112 (37.0)	85 (28.1)
Crude	1	0.639 (0.461, 0.884) **	0.624 (0.448, 0.871) **	0.463 (0.330, 0.649) **
Model 1	1	0.642 (0.445, 0.925) *	0.574 (0.393, 0.837) **	0.549 (0.376, 0.803) **
Model 2	1	0.607 (0.403, 0.915) *	0.557 (0.375, 0.827) **	0.515 (0.333, 0.796) **
Model 3	1	0.612 (0.406, 0.923) *	0.556 (0.374, 0.826) **	0.518 (0.335, 0.801) **

Model 1: adjusting for age, education, income, labor and exercise status. Model 2: Model 1 + dietary carbohydrate, fat, protein, fiber and cholesterol intake were adjusted. Model 3: Model 2 + smoking habits were adjusted. * *p* < 0.05; ** *p* < 0.01 compared with the 1st quartile.

**Table 4:** Multivariable-adjusted Odds ratio (OR) and 95% confidence interval (CI) of abdominal obesity by quartile of dietary histidine.

All				
Histidine Quartiles	Quartile 1	Quartile 2	Quartile 3	Quartile 4
Dietary histidine (% protein)	<1.38	1.38–1.59	1.59–1.77	>1.77
Participants, *n*	594	594	594	594
Abdominal obesity, *n* (%)	191 (32.1)	174 (29.3)	168 (28.3)	143 (24.1)
Crude	1	0.771 (0.603, 0.987) *	0.752 (0.587, 0.963) *	0.614 (0.477, 0.792) **
Model 1	1	0.733 (0.563, 0.956) *	0.774 (0.594, 0.996) *	0.742 (0.565, 0.974) *
Model 2	1	0.723 (0.545, 0.059) *	0.782 (0.579, 0.998) *	0.761 (0.552, 0.985) *
Model 3	1	0.716 (0.539, 0.952) *	0.809 (0.597, 0.995) *	0.754 (0.545, 0.943) *
**Men**				
**Histidine Quartiles**	**Quartile 1**	**Quartile 2**	**Quartile 3**	**Quartile 4**
Dietary histidine (% protein)	<1.35	1.35–1.57	1.57–1.76	>1.76
Participants, *n*	291	291	291	291
Abdominal obesity, *n* (%)	120 (41.2)	118 (40.5)	114 (39.2)	102 (35.1)
Crude	1	0.972 (0.698, 1.353)	0.918 (0.659, 1.279)	0.769 (0.550, 1.075)
Model 1	1	0.943 (0.663, 1.341)	0.923 (0.648, 1.315)	0.908 (0.634, 1.301)
Model 2	1	0.948 (0.650, 1.383)	0.940 (0.627, 1.410)	0.928 (0.600, 1.435)
Model 3	1	0.929 (0.636, 1.357)	0.959 (0.639, 1.441)	0.916 (0.592, 1.419)
**Women**				
**Histidine Quartiles**	**Quartile 1**	**Quartile 2**	**Quartile 3**	**Quartile 4**
Dietary histidine (% protein)	<1.40	1.40–1.61	1.61–1.77	>1.77
Participants, *n*	303	303	303	303
Abdominal obesity, *n* (%)	71 (23.4)	56 (18.5)	54 (17.8)	41 (13.5)
Crude	1	0.639 (0.461, 0.884) **	0.624 (0.448, 0.871) **	0.463 (0.330, 0.649) **
Model 1	1	0.499 (0.313, 0.794) **	0.539 (0.280, 0.938) *	0.507 (0.314, 0.816) **
Model 2	1	0.477 (0.293, 0.777) **	0.476 (0.278, 0.802) **	0.472 (0.272, 0.821) **
Model 3	1	0.478 (0.294, 0.778) **	0.501 (0.320, 0.840) **	0.473 (0.272, 0.822) **

Model 1: adjusting for age, education, income, labor and exercise status. Model 2: Model 1 + dietary carbohydrate, fat, protein, fiber and cholesterol intake were adjusted. Model 3: Model 2 + smoking habits were adjusted. *: *p* < 0.05; **: *p* < 0.01 compared with the 1st quartile.

**Table 5:** Associations between dietary histidine and metabolic profile, inflammation and oxidative stress in overweight/obese participants.

	Overall		Men		Women	
Parameters	*r* *	*p*	*r* *	*p*	*r* *	*p*
FBG	−0.179	<0.05	−0.171	<0.05	−0.214	<0.05
Insulin	−0.122	NS	−0.111	NS	−0.098	NS
HOMA-IR	−0.233	<0.05	−0.221	<0.05	−0.237	<0.05
TC	−0.103	NS	−0.089	NS	−0.109	NS
TG	−0.122	NS	−0.106	NS	−0.127	NS
HDL	0.074	NS	0.033	NS	0.098	NS
LDL	0.081	NS	0.089	NS	0.076	NS
2 h-PG	−0.171	<0.05	−0.167	NS	−0.189	<0.05
2 h-Insulin	−0.112	NS	−0.078	NS	−0.131	NS
2 h-TC	−0.081	NS	−0.065	NS	−0.057	NS
2 h-TG	−0.056	NS	−0.034	NS	−0.078	NS
2 h-HDL	0.098	NS	0.035	NS	0.101	NS
2 h-LDL	0.035	NS	0.008	NS	0.041	NS
GSH-Px	0.265	<0.05	0.231	<0.05	0.283	<0.05
SOD	0.167	<0.05	0.149	NS	0.198	<0.05
MDA	−0.202	<0.05	−0.187	<0.05	−0.231	<0.05
TNF-α	−0.271	<0.05	−0.265	<0.05	−0.273	<0.05
IL-1β	−0.178	<0.05	−0.172	<0.05	−0.189	<0.05
IL-6	−0.182	<0.05	−0.166	<0.05	−0.198	<0.05
CRP	−0.242	<0.05	−0.178	<0.05	−0.267	<0.05
Adiponectin	0.188	<0.05	0.176	<0.05	0.195	<0.05
Vaspin	−0.217	<0.05	−0.231	<0.05	−0.203	<0.05

Abbreviations: FBG, fasting blood glucose; HOMA-IR, homeostasis model assessment of insulin resistance; TC, total cholesterol; TG, triglyceride; HDL, high destiny lipoprotein; LDL, low destiny lipoprotein; 2 h-PG, 2 h-postprandial glucose; GSH-Px, glutathione peroxidase; SOD, superoxide dismutase; MDA, malonaldehyde; TNF-α, tumor necrosis factor-α; IL-1β, interleukin-1β; IL-6, interleukin-6; CRP, C-reactive protein; NS, no significance. * Adjusting for dietary carbohydrate, fat, protein, fiber and cholesterol intake.

## Supplementary Materials

The following are available online at http://www.mdpi.com/2072-6643/8/7/420/s1, Table S1: Characteristics of overall and subgroup participants.

## References

[1] Ng M., Fleming T., Robinson M., Thomson B., Graetz N., Margono C., Mullany E.C., Biryukov S., Abbafati C., Abera S.F. (2014). Global, regional, and national prevalence of overweight and obesity in children and adults during 1980–2013: A systematic analysis for the global burden of disease study 2013. Lancet.

[2] Kim S.K., Kim H.J., Hur K.Y., Choi S.H., Ahn C.W., Lim S.K., Kim K.R., Lee H.C., Huh K.B., Cha B.S. (2004). Visceral fat thickness measured by ultrasonography can estimate not only visceral obesity but also risks of cardiovascular and metabolic diseases. Am. J. Clin. Nutr..

[3] Wolongevicz D.M., Zhu L., Pencina M.J., Kimokoti R.W., Newby P.K., D’Agostino R.B., Millen B.E. (2010). Diet quality and obesity in women: The framingham nutrition studies. Br. J. Nutr..

[4] De Simone G., Devereux R.B., Chinali M., Roman M.J., Best L.G., Welty T.K., Lee E.T., Howard B.V. (2006). Strong Heart Study Investigators. Risk factors for arterial hypertension in adults with initial optimal blood pressure: The strong heart study. Hypertension.

[5] De Pergola G., Silvestris F. (2013). Obesity as a major risk factor for cancer. J. Obes..

[6] Hu F.B., Liu Y., Willett W.C. (2011). Preventing chronic diseases by promoting healthy diet and lifestyle: Public policy implications for China. Obes. Rev..

[7] Estruch R., Ros E., Martinez-Gonzalez M.A. (2013). Mediterranean diet for primary prevention of cardiovascular disease. N. Engl. J. Med..

[8] Choi J.H., Woo H.D., Lee J.H., Kim J. (2015). Dietary patterns and risk for metabolic syndrome in Korean women: A cross-sectional study. Medicine (Baltimore).

[9] Gardner C.D., Kiazand A., Alhassan S., Kim S., Stafford R.S., Balise R.R., Kraemer H.C., King A.C. (2007). Comparison of the atkins, zone, ornish, and learn diets for change in weight and related risk factors among overweight premenopausal women: The a to z weight loss study: A randomized trial. JAMA.

[10] Watanabe M., Suliman M.E., Qureshi A.R., Garcia-Lopez E., Barany P., Heimburger O., Stenvinkel P., Lindholm B. (2008). Consequences of low plasma histidine in chronic kidney disease patients: Associations with inflammation, oxidative stress, and mortality. Am. J. Clin. Nutr..

[11] Sun X., Feng R., Li Y., Lin S., Zhang W., Li Y., Sun C., Li S. (2014). Histidine supplementation alleviates inflammation in the adipose tissue of high-fat diet-induced obese rats via the nf-kappab- and ppargamma-involved pathways. Br. J. Nutr..

[12] Kasaoka S., Tsuboyama-Kasaoka N., Kawahara Y., Inoue S., Tsuji M., Ezaki O., Kato H., Tsuchiya T., Okuda H., Nakajima S. (2004). Histidine supplementation suppresses food intake and fat accumulation in rats. Nutrition.

[13] Niu Y.C., Feng R.N., Hou Y., Li K., Kang Z., Wang J., Sun C.H., Li Y. (2012). Histidine and arginine are associated with inflammation and oxidative stress in obese women. Br. J. Nutr..

[14] Feng R.N., Niu Y.C., Sun X.W., Li Q., Zhao C., Wang C., Guo F.C., Sun C.H., Li Y. (2013). Histidine supplementation improves insulin resistance through suppressed inflammation in obese women with the metabolic syndrome: A randomised controlled trial. Diabetologia.

[15] Okubo H., Sasaki S. (2005). Histidine intake may negatively correlate with energy intake in human: A cross-sectional study in Japanese female students aged 18 years. J. Nutr. Sci. Vitaminol. (Tokyo).

[16] Sublette M.E., Segal-Isaacson C.J., Cooper T.B., Fekri S., Vanegas N., Galfalvy H.C., Oquendo M.A., Mann J.J. (2011). Validation of a food frequency questionnaire to assess intake of n-3 polyunsaturated fatty acids in subjects with and without major depressive disorder. J. Am. Diet. Assoc..

[17] Haftenberger M., Heuer T., Heidemann C., Kube F., Krems C., Mensink G.B. (2010). Relative validation of a food frequency questionnaire for national health and nutrition monitoring. Nutr. J..

[18] Du S.S., Jiang Y.S., Chen Y., Li Z., Zhang Y.F., Sun C.H., Feng R.N. (2015). Development and applicability of an internet-based diet and lifestyle questionnaire for college students in china: A cross-sectional study. Medicine (Baltimore).

[19] Yingyangjiayuan. http://www.yyjy365.org/diet.

[20] Li Y.C., Li Y., Liu L.Y., Chen Y., Zi T.Q., Du S.S., Jiang Y.S., Feng R.N., Sun C.H. (2015). The ratio of dietary branched-chain amino acids is associated with a lower prevalence of obesity in young northern Chinese adults: An internet-based cross-sectional study. Nutrients.

[21] Yang Y., Wang G., Guo X. (2009). China Food Composition Tables.

[22] Zhou B.F. (2002). Effect of body mass index on all-cause mortality and incidence of cardiovascular diseases—Report for meta-analysis of prospective studies open optimal cut-off points of body mass index in Chinese adults. Biomed. Environ. Sci..

[23] Hu J., Wallace D.C., Jones E., Liu H. (2009). Cardiometabolic health of Chinese older adults with diabetes living in Beijing, China. Public Health Nurs..

[24] Matthews D.R., Hosker J.P., Rudenski A.S., Naylor B.A., Treacher D.F., Turner R.C. (1985). Homeostasis model assessment: Insulin resistance and beta-cell function from fasting plasma glucose and insulin concentrations in man. Diabetologia.

[25] De Moraes A.C., Bel-Serrat S., Manios Y., Molnar D., Kafatos A., Cuenca-Garcia M., Huybrechts I., Sette S., Widhalm K., Stehle P. (2015). Dietary protein and amino acids intake and its relationship with blood pressure in adolescents: The Helena study. Eur. J. Public Health.

[26] Kotsis V., Stabouli S., Papakatsika S., Rizos Z., Parati G. (2010). Mechanisms of obesity-induced hypertension. Hypertens. Res..

[27] Kahn S.E., Hull R.L., Utzschneider K.M. (2006). Mechanisms linking obesity to insulin resistance and type 2 diabetes. Nature.

[28] Greenberg A.S., Obin M.S. (2006). Obesity and the role of adipose tissue in inflammation and metabolism. Am. J. Clin. Nutr..

[29] Kern P.A., Ranganathan S., Li C., Wood L., Ranganathan G. (2001). Adipose tissue tumor necrosis factor and interleukin-6 expression in human obesity and insulin resistance. Am. J. Physiol. Endocrinol. Metab..

[30] Gonzalez A.S., Guerrero D.B., Soto M.B., Diaz S.P., Martinez-Olmos M., Vidal O. (2006). Metabolic syndrome, insulin resistance and the inflammation markers c-reactive protein and ferritin. Eur. J. Clin. Nutr..

[31] Lee Y.T., Hsu C.C., Lin M.H., Liu K.S., Yin M.C. (2005). Histidine and carnosine delay diabetic deterioration in mice and protect human low density lipoprotein against oxidation and glycation. Eur. J. Pharmacol..

[32] Son D.O., Satsu H., Shimizu M. (2005). Histidine inhibits oxidative stress- and tnf-alpha-induced interleukin-8 secretion in intestinal epithelial cells. FEBS Lett..

[33] Lihn A.S., Pedersen S.B., Richelsen B. (2005). Adiponectin: Action, regulation and association to insulin sensitivity. Obes. Rev..

[34] Hotta K., Funahashi T., Bodkin N.L., Ortmeyer H.K., Arita Y., Hansen B.C., Matsuzawa Y. (2001). Circulating concentrations of the adipocyte protein adiponectin are decreased in parallel with reduced insulin sensitivity during the progression to type 2 diabetes in rhesus monkeys. Diabetes.

[35] Hida K., Wada J., Eguchi J., Zhang H., Baba M., Seida A., Hashimoto I., Okada T., Yasuhara A., Nakatsuka A. (2005). Visceral adipose tissue-derived serine protease inhibitor: A unique insulin-sensitizing adipocytokine in obesity. Proc. Natl. Acad. Sci. USA.

[36] Youn B.S., Kloting N., Kratzsch J., Lee N., Park J.W., Song E.S., Ruschke K., Oberbach A., Fasshauer M., Stumvoll M. (2008). Serum vaspin concentrations in human obesity and type 2 diabetes. Diabetes.

[37] Feng R., Li Y., Wang C., Luo C., Liu L., Chuo F., Li Q., Sun C. (2014). Higher vaspin levels in subjects with obesity and type 2 diabetes mellitus: A meta-analysis. Diabetes Res. Clin. Pract..

[38] Ndisang J.F., Vannacci A., Rastogi S. (2014). Oxidative stress and inflammation in obesity, diabetes, hypertension, and related cardiometabolic complications. Oxid. Med. Cell. Longev..

[39] Kasaoka S., Kawahara Y., Inoue S., Tsuji M., Kato H., Tsuchiya T., Okuda H., Nakajima S. (2005). Gender effects in dietary histidine-induced anorexia. Nutrition.

[40] Nagai K., Tanida M., Niijima A., Tsuruoka N., Kiso Y., Horii Y., Shen J., Okumura N. (2012). Role of L-carnosine in the control of blood glucose, blood pressure, thermogenesis, and lipolysis by autonomic nerves in rats: Involvement of the circadian clock and histamine. Amino Acids.

[41] Courten B., Jakubova M., de Courten M.P., Kukurova I.J., Vallova S., Krumpolec P., Valkovic L., Kurdiova T., Garzon D., Barbaresi S. (2016). Effects of carnosine supplementation on glucose metabolism: Pilot clinical trial. Obesity (Silver Spring).

