# Supplementary material for: Relationships of Dietary Histidine and Obesity in Northern Chinese Adults, an Internet-Based Cross-Sectional Study
Source: Nutrients. 2016 Jul 11;8(7):420. doi: 10.3390/nu8070420 (PMC4963896; doi:10.3390/nu8070420)
Supplement: Supplementary file 1 [file nutrients-08-00420-s001.docx]

Supplementary Materials: Relationships of Dietary Histidine and Obesity in Northern Chinese Adults, an Internet-Based Cross-Sectional Study

Yan-Chuan Li, Chun-Long Li, Jia-Yue Qi, Li-Na Huang, Dan Shi, Shan-Shan Du, Li-Yan Liu, Ren-Nan Feng and Chang-Hao Sun

CV of Lab Measurements

For the biochemical analyses, the intra- and inter-assay coefficients of variations (CV%) of different parameters were listed as follows: serum glucose (intra-day CV%, 4.38%; inter-day CV%, 6.85%); total cholesterol (intra-day CV%, 4.16%; inter-day CV%, 6.27%); triglyceride (intra-day CV%, 4.94%; inter-day CV%, 7.03%); high density lipoprotein cholesterol (intra-day CV%, 4.85%; inter-day CV%, 6.85%); low density lipoprotein cholesterol (intra-day CV%, 4.53%; inter-day CV%, 6.16%); insulin (intra-day CV%, 4.31%; inter-day CV%, 5.27%); TNF-α, IL-1β, IL-6, and vaspin (intra-day CV%, <8%; inter-day CV%, <10%); C-reactive protein (intra- and inter-day CV%, <20%); and adiponectin (intra-day CV%, <10%; inter-day CV%, <12%).

**Table S1.** Characteristics of overall and subgroup participants.

|  | **Overall** | **Subgroup** | ***p*** |
| --- | --- | --- | --- |
| Participants, *n* | 2376 | 88 |  |
| Histidine, % total protein intake | 1.56 ± 0.3 | 1.42 ± 0.2 | <0.05 |
| Age, year | 33.1 ± 15.4 | 33.9 ± 12.6 | NS |
| Gender |  |  | NS |
| Men, *n* (%) | 1164 (49.0) | 44 (50.0) |  |
| Women, *n* (%) | 1212 (51.0) | 44 (50.0) |  |
| Body weight, kg | 65.8 ± 11.7 | 77.9 ± 10.1 | <0.01 |
| BMI, kg/m^2^ | 23.3 ± 3.4 | 27.6 ± 1.9 | <0.001 |
| WC, cm | 79.2 ± 9.9 | 89.5 ± 8.1 | <0.001 |
| Income per month |  |  | NS |
| <2000 yuan, *n* (%) | 1537 (64.7) | 55 (62.5) |  |
| 2000–5000 yuan, *n* (%) | 756 (31.8) | 27 (30.7) |  |
| ≥5000 yuan, *n* (%) | 83 (3.5) | 6 (6.8) |  |
| Education |  |  | NS |
| Under college, *n* (%) | 574 (24.2) | 23 (26.1) |  |
| Bachelor, *n* (%) | 1708 (71.9) | 64 (72.7) |  |
| Master or doctor, *n* (%) | 94 (4.0) | 1 (1.1) |  |
| Labor |  |  | NS |
| Light, *n* (%) | 627 (26.4) | 27 (30.7) |  |
| Medium, *n* (%) | 1671 (70.3) | 61 (69.3) |  |
| Heavy, *n* (%) | 78 (3.3) | 0 (0) |  |
| Exercise |  |  | NS |
| <10 h/week, *n* (%) | 823 (34.6) | 34 (38.6) |  |
| 10–20 h/week, *n* (%) | 1266 (53.3) | 46 (52.3) |  |
| ≥20 h/week, *n* (%) | 287 (12.1) | 8 (9.1) |  |
| Smoking |  |  | <0.001 |
| Non-smoker, *n* (%) | 2024 (85.2) | 88 (100.0) |  |
| Current smoker, *n* (%) | 258 (10.9) | 0 (0) |  |
| Quit smoking, *n* (%) | 94 (4.0) | 0 (0) |  |
| Drinking |  |  | <0.001 |
| Non-drinker, *n* (%) | 1939 (81.6) | 88 (100.0) |  |
| Current drinker, *n* (%) | 437 (18.4) | 0 (0) |  |
| SBP (mmHg) | 117.7 ± 12.8 | 124.0 ± 10.2 | <0.05 |
| DBP (mmHg) | 77.9 ± 8.8 | 81.1 ± 7.8 | <0.05 |
| Dietary intakes |  |  |  |
| Energy, kcal/day | 2453.3 ± 864.7 | 2489.4 ± 666.8 | NS |
| Total protein, g/day | 90.3 ± 37.0 | 92.1 ± 32.9 | NS |
| Total amino acids, g/day | 55.5 ± 26.6 | 56.9 ± 25.8 | NS |
| Histidine, g/day | 1.4 ± 0.7 | 1.3 ± 0.6 | NS |
| Total fat, g/day | 75.9 ± 37.5 | 76.7 ± 34.6 | NS |
| Total carbohydrate, g/day | 369.1 ± 142.4 | 375.1 ± 136.7 | NS |
| Cholesterol, mg/day | 456.8 ± 329.3 | 461.5 ± 336.2 | NS |
| Fiber, g/day | 20.1 ± 11.1 | 19.1 ± 10.2 | NS |

Abbreviations: BMI, body mass index; WC, waist circumference; SBP, systolic blood pressure; DBP, diastolic blood pressure; NS, no significance. Data are expressed as mean ± SD, frequencies and percentages, as appropriate.
